# Labor epidural analgesia and risk of autism Spectrum disorders in offspring: A systematic review and meta-analysis

**DOI:** 10.3389/fped.2022.965205

**Published:** 2023-02-20

**Authors:** Ling-ling Fang, Yuan-yue Zhou, Hai-yin Jiang, Yu-dan Shi

**Affiliations:** ^1^ Department of Anesthesiology, the Second Affiliated Hospital, School of Medicine, Zhejiang University, Hangzhou, China; ^2^ Department of Child Psychiatry, Hangzhou Seventh People's Hospital, Hangzhou, China; ^3^ Collaborative Innovation Center for Diagnosis and Treatment of Infectious Diseases, State Key Laboratory for Diagnosis and Treatment of Infectious Diseases, the First Affiliated Hospital, School of Medicine, Zhejiang University, Hangzhou, China; ^4^ Department of Chinese Internal Medicine, Taizhou First People's Hospital, Taizhou, China

**Keywords:** neurodevelopment, pain, children, meta-analysis, analgesia

## Abstract

**Background:**

The effect of labor epidural anesthesia (LEA) on the risk of autism spectrum disorder (ASD) in offspring has been investigated recently, and available results are inconsistent.

**Methods:**

We searched the PubMed and EMBASE databases for relevant studies and performed a systematic review and meta-analysis of the literature. Subgroup analyses were conducted to assess the sources of heterogeneity. Both fixed and random effects models were used was used to estimate overall relative risk.

**Results:**

Our results showed that LEA was associated with an increased risk of ASD in offspring [HR = 1.3, 95% confidence interval (CI): 1.25–1.35; *P* < 0.001] after combining crude estimates from the included studies. This association was gradually reduced, but still statistically significant, when potential confounding factors were considered (HR 1.13, 95% CI 1.03–1.25, *P* = 0.014). However, there was no significant association when we combined data of siblings from other pregnancies (HR = 1.07, 95% CI: 0.99–1.16, *P* = 0.076), implying that the association was due to confounding factors.

**Conclusion:**

The statistically significant association between LEA and ASD in the offspring can be partially explained by unmeasured confounding.

**Systematic Review Registration:**

Identifier CRD42022302892.

## Introduction

Autism spectrum disorder (ASD) is a heterogeneous neurodevelopmental disorder characterized by deficits in social communication and social interaction and the presence of restricted, repetitive behaviors (1). The worldwide population prevalence is about 1% (1). Over the past decade, the incidence of ASD has dramatically increased (2). Although ASD is highly heritable, environmental factors have been shown to be involved in the development of this disorder (3). Thus, recognition of the risk factors for ASD and implementation of appropriate interventions may help to prevent the disorder.

Labor epidural anesthesia (LEA) is the most popular method of pain relief during labor (4). In recent years, growing numbers of women have received some form of neuraxial procedure during labor (5). Although the effectiveness and safety of LEA for the fetus and newborn have been well described (6), the long-term effects of LEA on the offspring remain unclear. Preclinical studies have demonstrated that standard clinical doses of local anesthetics can alter the normal course of behavioral development in rhesus monkeys (7). Observational studies found that only Cesarean section performed with general anesthesia was associated with an increased risk of ASD compared with vaginal deliveries (8, 9). However, these studies did not evaluate the potential risk associated with the common use of neuraxial anesthesia for routine vaginal delivery. Recently, several epidemiological studies (10–14) have investigated the contribution of LEA to the risk of ASD with varying results. In the earliest study, Qiu et al. (10) reported that LEA was still associated with an increased risk of ASD after taking epidural-related maternal fever into consideration. Meanwhile, one study (11) in Canada also found a significant association between LEA and the risk of ASD in offspring. However, this association was not observed in the latest three studies (12–14). Given that LEA is currently the criterion standard for labor pain management during routine vaginal delivery, it is important to determine whether there is a relation between LEA and the risk of ASD in offspring. We conducted a systematic literature review and meta-analysis to assess the association between fetal exposure to LEA and the subsequent development of ASD.

## Methods

This meta-analysis was conducted according to the PRISMA (Preferred Reporting Items of Systematic Reviews and Meta-analysis) guidelines (15). We pre-registered the protocol with PROSPERO (CRD42022302892).

### Search strategy

Using the Embase and PubMed Databases, we conducted a search for all studies published in English until January 26, 2022. The search was performed using the terms “labour OR labor” AND “anesthesia OR analgesia” AND “Autism Spectrum Disorder OR Autism OR ASD”. To ensure a complete review of the available studies, reference lists of relevant published literature were manually checked to identify additional eligible meta-analyses.

### Study selection

Two of the authors (LLF and HYJ) independently evaluated the eligibility of all relevant articles based on the selection criteria until January 28, 2022. Full texts were retrieved after reading the titles and abstracts. Any discrepancies were resolved by discussion with a third author (YYZ). Peer-reviewed studies were included if they met the following (PICO) criteria: (1) types of studies: randomized controlled trials (RCTs), cohort, nested case–control, and case–control studies; (2) type of participant: children exposed or unexposed to LEA; (3) type of intervention: LEA administered during labor and delivery with a valid control group who received no LEA during labor and delivery; (4) types of outcome measures: subsequent ASD development reported in studies with the adjusted ORs or RRs or HRs and 95% confidence intervals (CIs) or provision of adequate data to calculate risk estimates.

### Data extraction and quality assessment

Data were extracted independently by YYZ and YDS, and discrepancies were resolved by a third author (HYJ) before the final analysis. The following data were extracted: author, year of publication, data source, study time/period, study design, number of participants, outcome assessment, ascertainment of LEA exposure, and study quality. We assessed the methodologic quality of the included studies using the Newcastle–Ottawa Scale (NOS) as recommended by the Cochrane Collaboration (16). A score >7 points was taken to indicate a high-quality study.

### Statistical analysis

All data management and analyses were performed using Stata SE software (ver. 13.0; StataCorp, College Station, TX, USA). Random effects models were used to analyse pooled effects when statistical heterogeneity existed. Otherwise, fixed effects models were used (17). The *I*^2^ statistic was used to assess between-study heterogeneity; studies with *I*^2^ values <25% were considered minimal heterogeneous, values between 25% and 50% indicated moderate heterogeneity, and values ≥50% indicated statistical heterogeneity (18). Publication bias was not assessed because the meta-analysis included fewer than 10 studies (19, 20). All statistical analyses were two-sided, and *p*-values < 0.05 were considered statistically significant.

## Results

### Search results

**Figure 1:**
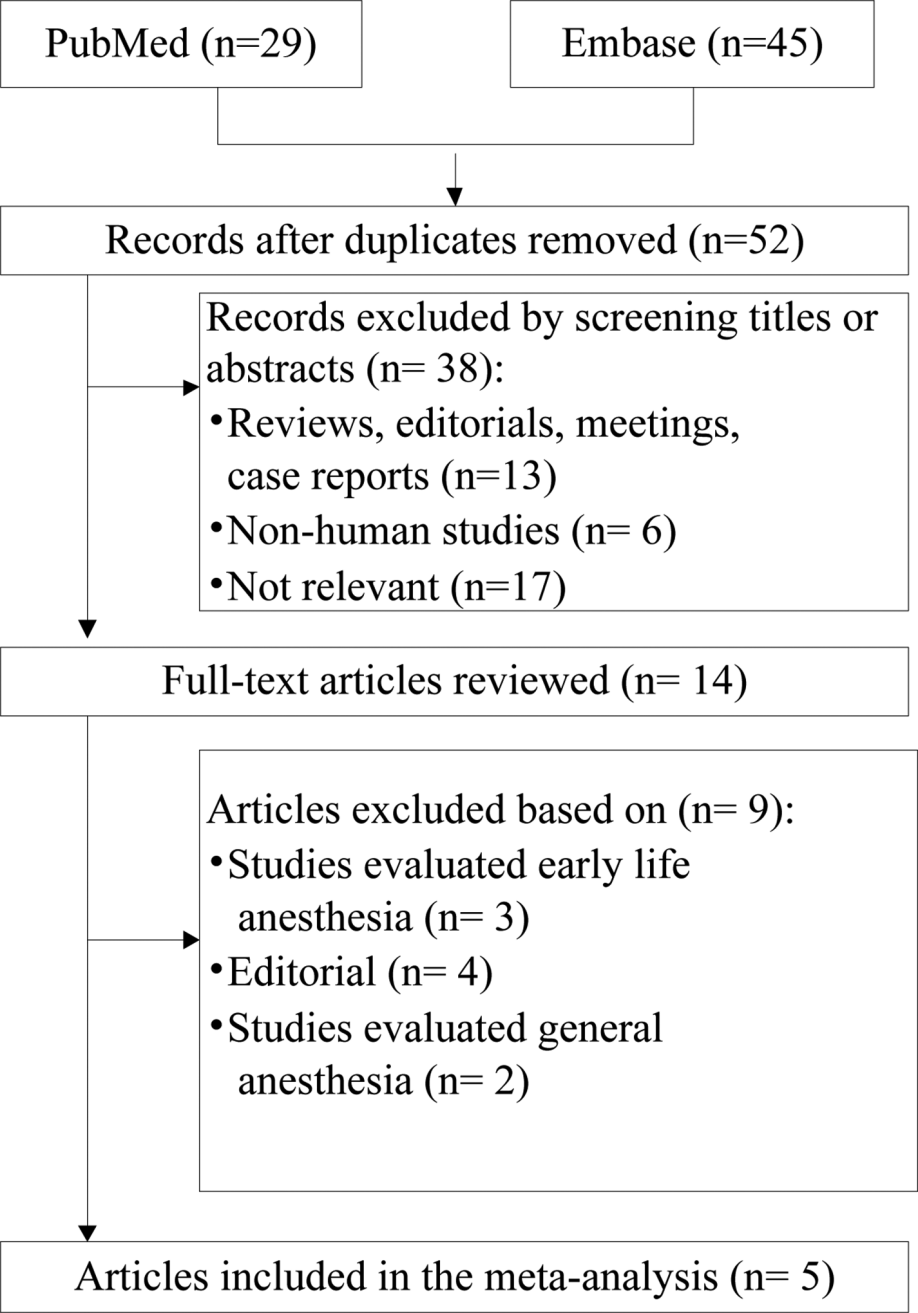
Flow chart of the studies considered and finally selected for review.

This systematic review identified 74 references from these two databases. After adjusting for duplicates, a total of 52 papers were entered into full-text review, with 38 excluded immediately on inspection of the title and abstract. Two studies (12, 14) used data from the Danish Medical Birth Register. Although the study period of Ren et al. fully covered that of Mikkelsen et al., Mikkelsen et al. conducted further analyses to test robustness of the overall analysis; hence, Mikkelsen et al.'s study (12) was included in the subgroup-analysis. Finally, five cohort studies (10–14) were identified for inclusion in the review. Some of the excluded studies, together with the reasons for their exclusion, are presented in Figure 1.

### Characteristics of the included studies

Characteristics of the five studies are presented in Table 1. All included studies were published in the past year, and all had large sample sizes, ranging from 123,175 to 624,952. Two studies (11, 13) were performed in Canada, one (10) in the USA, and the remaining two (12, 14) in Denmark. Exposure to LEA was assessed using pharmacy data, and valid diagnostic definitions of ASD were used to identify ASD cases in all studies. The extent of adjustment for potential clinical risk factors varied considerably across studies. Based on the methodological quality assessment scores, all studies were of high quality; their mean score was 8.8. The breakdown of scores is shown in Supplementary Table S1.

**Table 1:** Characteristics of the included studies.

Author, year	Location, setting	Study design/ Born period	LEA measurement	ASD assessment	Age at ASD assessment	Age range at the end of study	Number of children	HR, 95%CI				Quality
								Crude	Adjusted^a^	Adjusted^b^	Fully adjusted^c^	
Qiu et al, 2020	USA, population-based	Retrospective cohort study, 2008-2015	Pharmacy data	ICD-9	1.5	3 -11	Unexposed: 485/38,176 Exposed: 2,039/109,719	Overall: 1.48 (1.34-1.65)	NA	NA	Overall: 1.37 (1.22-1.53) Full term: 1.4 (1.25-1.57)	8
Wall-Wieler et al, 2021	Canada (Manitoba), population-based	Retrospective cohort study, 2005-2016	Pharmacy data	ICD-9 or ICD-10	1.5	4.5–15.5	Unexposed: 1,272/76,164 Exposed: 985/47,011	Overall: 1.25 (1.15-1.36)	Overall: 1.28 (1.17-1.4)	Overall: 1.15 (1.04-1.26)	Overall: 1.08 (0.97-1.2) Full term: 1.09 (0.98-1.22) Sibling: 0.97 (0.78-1.22) Restrictive definition: 1.04 (0.91-1.2) First birth only: 1.03 (0.88-1.2)	9
Hanley et al, 2021	Canada (British Columbia), population-based	Retrospective cohort study, 2000-2014	Pharmacy data	ADOS or ADOS and ADI-R	2	2–16.5	Unexposed: 3,482/276,774 Exposed: 1,710/111,480	Overall: 1.32 (1.24-1.4)	Overall: 1.3 (1.22-1.38)	Overall: 1.12 (1.05-1.2)	Overall: 1.09 (1-1.15) Sibling: 1.1 (0.99-1.2)	9
Mikkelsen et al, 2021	Denmark, population-based	Retrospective cohort study, 2006-2013	Pharmacy data	ICD-10	1	4–12	Unexposed: 5,019/386,278 Exposed: 1,409/92,990	Overall: 1.29 (1.21-1.37)	NA	NA	Overall: 1.05 (0.98-1.11) Full term: 1.05 (0.98-1.12) Sibling: 1.05 (0.9-1.21) Restrictive definition: 1.05 (0.96-1.14) First birth only: 1.06 (0.99-1.14)	9
Ren et al, 2021	Denmark, population-based	Retrospective cohort study, 2005-2016	Pharmacy data	ICD-10	1	2 -14	Unexposed: 6,023/508,656 Exposed: 1,648/116,296	Overall: 1.38 (1.31-1.46)	NA	NA	Overall: 1.11 (1.04-1.18) Sibling: 1.03 (0.84-1.27)	9

ADI-R, Autism Diagnostic Interview–Revised; ADOS, Autism Diagnostic Observation Schedule; NA, not available; ICD, International Classification Of Diseases.

^a^ Adjusted for maternal sociodemographic covariates.

^b^ Adjusted for maternal sociodemographic, pre-pregnancy and pregnancy-related covariates.

^c^ Adjusted for maternal sociodemographic, pre-pregnancy and pregnancy-related, and perinatal covariates.

### Meta-analysis

**Figure 2:**
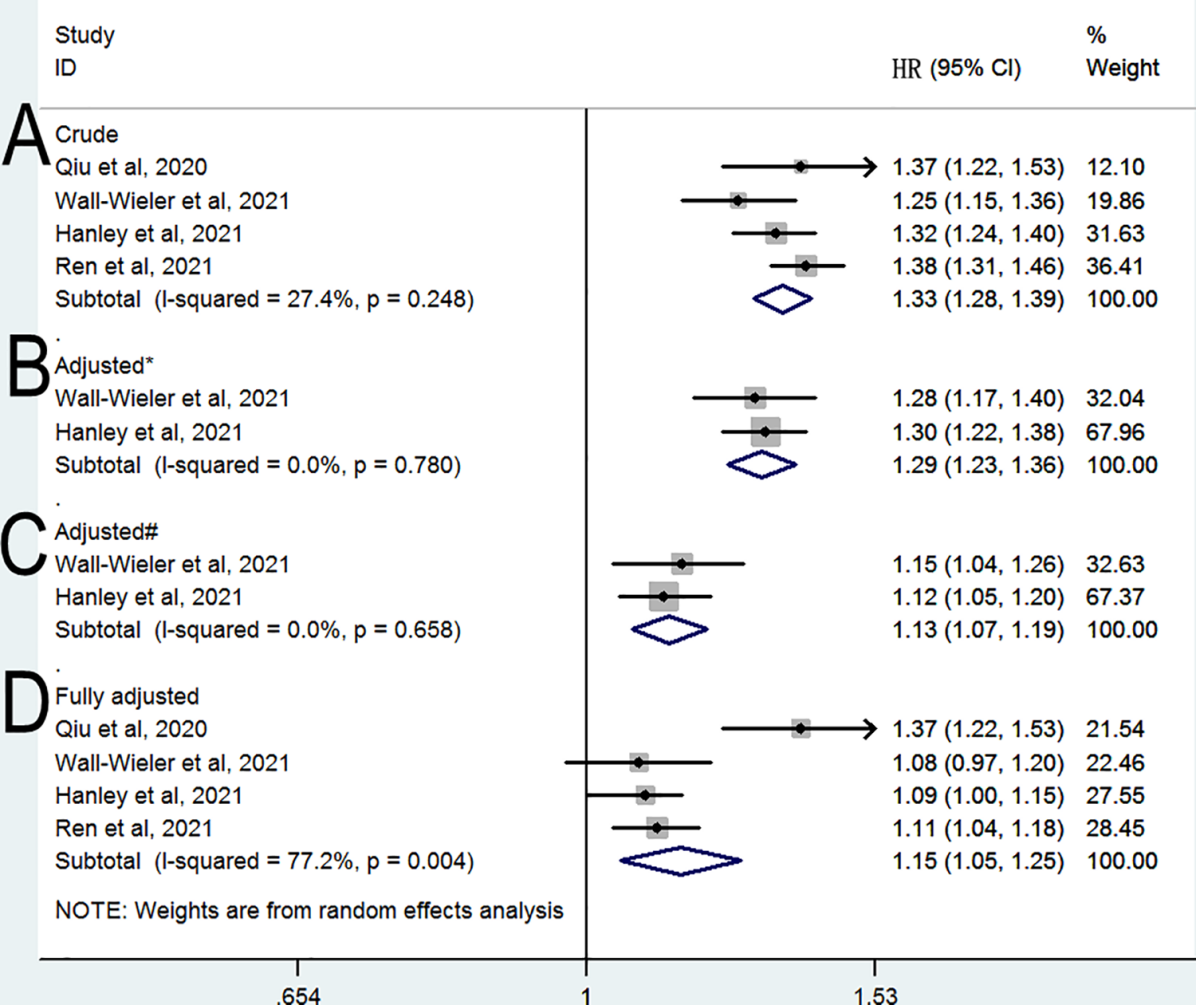
Relative risk of subsequent ASD (**A**) crude HR (**B**) HR adjusted for * (**B**) HR adjusted for ^#^ (**B**) fully & adjusted HR. * Adjusted for maternal sociodemographic covariates; ^#^ Adjusted for maternal sociodemographic, pre-pregnancy and pregnancy-related covariates; ^&^Adjusted for maternal sociodemographic, pre-pregnancy and pregnancy-related, and perinatal covariates.

The meta-analysis of the four cohort studies (10, 11, 13, 14) revealed a significant relation between LEA exposure and the risk of ASD (HR 1.33, 95% CI 1.28–1.39, *P* < 0.001; Figure 2A) after combing the crude estimates; furthermore, we found moderate heterogeneity across the studies (*I*^2^ = 27.4%). When the analysis was limited to two studies (11, 13) adjusted for only maternal sociodemographic covariates, the pooled HR was 1.29 (95% CI: 1.23–1.36, *P* < 0.001; *I*^2^ = 0%; Figure 2B). When the analysis was limited to two studies (11, 13) that were adjusted for maternal pre-pregnancy and pregnancy related covariates, the pooled HR was 1.13 (95% CI: 1.07–1.19, *P* < 0.001; *I*^2^ = 0%; Figure 2C). The meta-analysis of the four cohort studies (10, 11, 13, 14) revealed a significant relation between LEA exposure and the risk of ASD (HR 1.15, 95% CI 1.05–1.25, *P* = 0.002; Figure 2D) when combining the fully adjusted estimates; however, we found significant heterogeneity across the studies (*I*^2^ = 82.1%). When the analysis was limited to three studies included children older than 12 years at the end of study, the pooled HR was 1.1 (95% CI: 1.05–1.15, *P* < 0.001; *I*^2^ = 0%).

**Figure 3:**
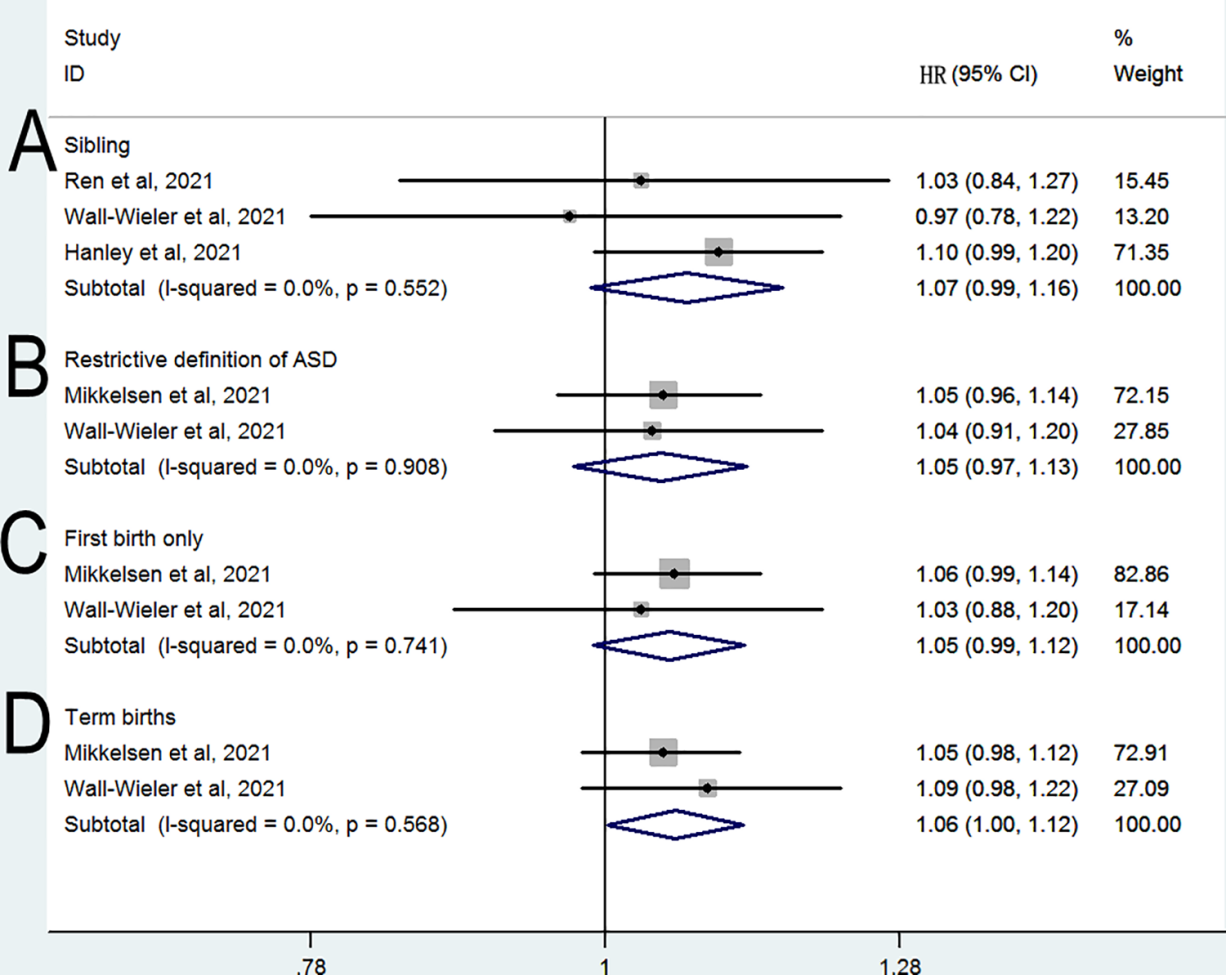
Relative risk of subsequent ASD in subgroup analyses (**A**) sibling (**B**) restrictive definition of ASD (**C**) first birth only (**D**) term births.

A sibling-matched analysis was conducted in three studies (11, 13, 14) to control for confounding genetic and social factors. As shown in Figure 3A, this analysis revealed a nonsignificant difference in the risk of ASD between siblings who were and those who were not exposed to LEA (HR = 1.07, 95% CI: 0.99–1.16, *P* = 0.098; *I*^2^ = 0%). When the analysis was limited to two studies (12, 13) with restrictive definitions of ASD, no significant difference was observed in the risk of ASD (HR = 1.05, 95% CI: 0.97–1.13, *P* = 0.215; *I*^2^ = 0%; Figure 3B). When the analysis was limited to two studies (12, 13) evaluating first birth only, no significant difference was observed in the risk of ASD (HR = 1.05, 95% CI: 0.99–1.12, *P* = 0.103; *I*^2^ = 0%; Figure 3C). When the analysis was limited to two studies (12, 13) evaluating term birth only, a significant difference was observed in the risk of ASD (HR = 1.06, 95% CI: 1–1.12, *P* = 0.043; *I*^2^ = 0%; Figure 3D).

## Discussion

Our findings indicate that LEA exposure was associated with a subsequent risk of ASD in the offspring after combining the crude data. This association was gradually reduced, but still statistically significant, when potential confounding factors were considered step by step. However, results from restrictive definitions of ASD and term birth only suggest that LEA use is not associated with an increased offspring risk of ASD. Furthermore, the sibling-matched analysis showed a nonsignificant effect toward an increased risk of ASD, indicating that genetic and familial confounding factors may largely explain the observed association. Because our review included only a small number of studies, the results should be interpreted with caution.

Our main analysis, based on four observational studies (10, 11, 13, 14), was limited by the existence of residual unknown confounders. All four studies found a positive association between maternal LEA exposure and ASD in the unadjusted model; a significant increased risk of ASD (pooled HR = 1.33) was also observed after we pooled the crude estimates from the included studies. Maternal age, parents' educational background, and economic status were associated with ASD development in the offspring (3); when combined with the estimates adjusted for maternal sociodemographic factors, the risk (pooled HR = 1.29) was comparable to the pooled crude HR. Considering the role of other environmental factors in offspring ASD, two studies (11, 13) gradually added pregnancy-related and perinatal factors and in their adjusted models; the pooled adjusted HR was reduced to 1.13 and 1.15, suggesting that any observed association could be partially explained by potential confounding factors. Also, previous epidemiological studies (21–23) found that a family history of ASD and psychiatric diseases was strongly associated with an increased risk of ASD in the offspring. The study conducted by Qiu et al. (10) reported the highest risk of ASD (adjusted HR = 1.37) among the included studies. However, their findings did not consider the history of mental disorder, and the prevalence of mental disorders was higher in mothers exposed to LEA; thus, the association may be overestimated in this study. Therefore, the ideal control for the unmeasured confounding factors would be a sibling-matched design, which should minimize the effects of familial factors on the observed association. Our analysis based on a sibling-matched design found that the relationship between exposure to LEA and ASD was not statistically significant, suggesting that any observed association could be a result of genetic factors. It also should be noted that the heterogeneity among the three sibling-matched studies (11, 13, 14) was reduced to 0%. The sample sizes in the sibling-matched studies were small, and further studies are needed to verify these results.

In our main analysis based on fully adjusted estimates, we observed high heterogeneity among the included studies. To explore the clinical heterogeneity and test the robustness of our results, we conducted further subgroup analyses. The studies used various forms of assessment for ASD and different diagnostic definitions of ASD, which could lead to substantially different assessments even in the same study population. To minimize heterogeneity, subgroup analyses based on a restrictive definition of ASD were performed; these found no significant increase in the risk of ASD. Meanwhile, an analysis limited to studies that provided data for first-birth offspring found no difference in ASD risk between children exposed and those unexposed to LEA. This may result for two reasons. First, their sample sizes are small, and their confidence intervals are large. Hence, the results of those studies are inconclusive. Second, it could be that first born individuals are less susceptible to possible adverse effects of LEA. A previous meta-analysis (24) demonstrated that preterm birth was associated with an increased risk of ASD, and three studies (10, 12, 13) included in the present analysis that provided data on term birth revealed a small but significant increase in the risk of ASD (pooled HR = 1.06). The results of our subgroup analyses may be limited by sample size, and further investigation is needed to clarify the effects of these factors on the risk of ASD.

This systematic review and meta-analysis is the first to provide an overall estimate of the effect of maternal LEA exposure on ASD risk in offspring. The strength of our meta-analysis lies in the exclusive use of cohort studies, which are less prone to bias in terms of assessing LEA exposure. In addition, the included studies were of high quality and used valid assessments to evaluate ASD. Another strength of this meta-analysis is the careful consideration of potential confounding factors, especially in step-by-step analyses including the adjustments for confounding factors and subgroup analyses based on sibling-matched studies.

Nevertheless, the study has several limitations. First, the number of included studies in which ASD risk was evaluated was small, especially for sub-group analyses. Second, all reviewed studies were performed with European and North American populations with no subjects from Asian or African countries, which may have affected the generalizability of our findings. Third, limited data were available on the duration of LEA in the included studies; therefore, we could not draw robust conclusions about exposure parameters potentially associated with ASD risk. Finally, controlling confounders in observational studies is a major challenge for causal inference. Future well-designed studies using methods of causal inference (e.g., the use of natural experiments or sensitivity analysis) and considering the duration of LEA are needed to clarify the contribution of LEA to the risk of ASD in children.

Current evidence suggests the associations between LEA and ASD risk in the offspring may be overestimated because previous studies failed to control for genetic confounding factors. Therefore, our findings might not warrant a recommendation to prohibit LEA used pain relief during labor and delivery. Meanwhile, children exposed to LEA do not require additional ASD surveillance.

## Conclusion

In conclusion, the findings of our meta-analysis suggest a small but significant link between LEA and ASD risk in the offspring. However, we could not exclude the possibility that this association was overestimated due to potential residual confounders.

## Data Availability

The original contributions presented in the study are included in the article/**Supplementary Material**, further inquiries can be directed to the corresponding author/s.
