# Supplementary material for: Labor epidural analgesia and risk of autism Spectrum disorders in offspring: A systematic review and meta-analysis
Source: Front Pediatr. 2023 Feb 20;10:965205. doi: 10.3389/fped.2022.965205 (PMC9986298; doi:10.3389/fped.2022.965205)
Supplement: Supplementary file 2 [file Table1.docx]

| **Table S1**  NOS for Assessment of Quality of Included Studies: Cohort Studies | | | | | | | | |
| --- | --- | --- | --- | --- | --- | --- | --- | --- |
| Study | Selection | | | | Comparability | | Outcomes | |
|  | Representativeness of exposed cohort? | Selection of the nonexposed cohort? | Ascertainment of exposure? | Demonstration that outcome of interest was not represent at the start of the study | Comparability of Cohort* | Assessment of outcome | Was follow-up long enough for outcomes to occur | Adequacy of follow up of cohorts |
| Qiu et al, 2020 | ★ | ★ | ★ | ★ | ★ | ★ | ★ | ★ |
| Wall-Wieler et al, 2021 | ★ | ★ | ★ | ★ | ★★ | ★ | ★ | ★ |
| Hanley et al, 2021 | ★ | ★ | ★ | ★ | ★★ | ★ | ★ | ★ |
| Mikkelsen et al, 2021 | ★ | ★ | ★ | ★ | ★★ | ★ | ★ | ★ |
| Ren et al, 2021 | ★ | ★ | ★ | ★ | ★★ | ★ | ★ | ★ |
| Note: A star denotes a score of 1; * A maximum of 2 stars can be allotted in this category | | | | | | | | |
